# The SpO_2_/FiO_2_ Ratio Combined with Prognostic Scores for Pneumonia and COVID-19 Increases Their Accuracy in Predicting Mortality of COVID-19 Patients

**DOI:** 10.3390/jcm13195884

**Published:** 2024-10-02

**Authors:** Giuseppe Zinna, Luca Pipitò, Claudia Colomba, Nicola Scichilone, Anna Licata, Mario Barbagallo, Antonio Russo, Nicola Coppola, Antonio Cascio

**Affiliations:** 1 Department of Surgery, Dentistry, Paediatrics, and Gynaecology, Division of Cardiac Surgery, University of Verona Medical School, 37129 Verona, Italy; giuseppe.zinna@studenti.univr.it; 2 Department of Health Promotion, Mother and Child Care, Internal Medicine and Medical Specialties, University of Palermo, 90127 Palermo, Italy; luca.pipito@community.unipa.it (L.P.); claudia.colomba@unipa.it (C.C.); nicola.scichilone@unipa.it (N.S.); anna.licata@unipa.it (A.L.); mario.barbagallo@unipa.it (M.B.); 3 Pediatric Infectious Disease Unit, “G. Di Cristina” Hospital, ARNAS Civico Di Cristina Benfratelli, 90127 Palermo, Italy; 4 Section of Infectious Diseases, Department of Mental Health and Public Medicine, University of Campania “Luigi Vanvitelli”, 80131 Naples, Italy; antonio.russo2@unicampania.it (A.R.); nicola.coppola@unicampania.it (N.C.); 5 Infectious and Tropical Disease Unit, AOU Policlinico “P. Giaccone”, 90127 Palermo, Italy

**Keywords:** COVID-19, SpO_2_, PaO_2_/FiO_2_ ratio, SpO_2_/FiO_2_ ratio, prognostic score, risk score, PSI, CRB-65, CZ COVID-19

## Abstract

**Background:** Identifying high-risk COVID-19 patients is critical for emergency department decision-making. Our study’s primary objective was to identify new independent predictors of mortality and their predictive utility in combination with traditional pneumonia risk assessment scores and new risk scores for COVID-19 developed during the pandemic. **Methods**: A retrospective study was performed in two Italian University Hospitals. A multivariable logistic model was used to locate independent parameters associated with mortality. **Results:** Age, PaO_2_/FiO_2_, and SpO_2_/FiO_2_ ratios were found to be independent parameters associated with mortality. This study found that the Pneumonia Severity Index (PSI) was superior to many of the risk scores developed during the pandemic, for example, the International Severe Acute Respiratory Infection Consortium Coronavirus Clinical Characterisation Consortium (ISARIC 4C) (AUC 0.845 vs. 0.687, *p* < 0.001), and to many of the risk scores already in use, for example, the National Early Warning Score 2 (NEWS2) (AUC 0.845 vs. 0.589, *p* < 0.001). Furthermore, our study found that the Pneumonia Severity Index had a similar performance to other risk scores, such as CRB-65 (AUC 0.845 vs. 0.823, *p* = 0.294). Combining the PaO_2_/FiO_2_ or SpO_2_/FiO_2_ ratios with the risk scores analyzed improved the prognostic accuracy. **Conclusions:** Adding the SpO_2_/FiO_2_ ratio to the traditional, validated, and already internationally known pre-pandemic prognostic scores seems to be a valid and rapid alternative to the need for developing new prognostic scores. Future research should focus on integrating these markers into existing pneumonia scores to improve their prognostic accuracy.

## 1. Introduction

Noninvasive risk assessment is crucial in patients with COVID-19 in the emergency department [1]. Objective prognostic information describing the probability of survival for patients on admission may inform doctors in improving triage before admission and the proportionality of treatment during admission [2]. There have been efforts to develop predictive scores and algorithms to address these needs, and an example is the International Severe Acute Respiratory Infection Consortium Coronavirus Clinical Characterisation Consortium (ISARIC 4C) [3]. Given the heterogeneity in symptoms on presentation and the potential outcomes [4,5], it is not surprising that many of the tools proposed so far have relied on complex sets of parameters or specialized laboratory markers. New prognostic scores created during the COVID-19 pandemic have been added to the old prognostic scores for pneumonia, such as the Pneumonia Severity Index (PSI), CURB-65, and NEWS2. The PSI has been demonstrated to be a powerful tool for predicting mortality in patients with COVID-19, and it performed significantly better than CURB-65 [6]. The simplified score of the CURB-65 or CRB-65 has been used to assess the severity of COVID-19 without the limit of laboratory data for blood urea nitrogen, especially during the COVID-19 pandemic; which may be a helpful score tool because of its simplicity in application, especially in emergent and complicated conditions [7]. Also, the National Early Warning Score 2 (NEWS2) index has been used to identify the risk of death of COVID-19 patients [8].

The PaO_2_/FiO_2_ ratio, derived from arterial blood gas analysis, remains the gold standard for diagnosing acute respiratory failure. The noninvasive SpO_2_/FiO_2_ ratio has been found to correlate well with the PaO_2_/FiO_2_ ratio in adult and pediatric patients with pneumonia, acute respiratory distress syndrome (ARDS), and acute lung injury in various studies [9,10,11]. The SpO_2_/FiO_2_ ratio, measured within the first 6 h after hospital admission, is an independent indicator of ARDS development among patients at risk [12]. The SpO_2_/FiO_2_ ratio could represent a diagnostic and prognostic marker as a potential substitute, especially in resource-limited settings [13]. The ABC2-SPH risk score is an easy-to-use rapid scoring system based on the characteristics of COVID-19 patients, commonly available at hospital admission. It was designed and validated for early stratification of in-hospital mortality risk of patients with COVID-19 [14,15]. An analogous mortality risk score is the COVID-19 MRS, in which the PaO_2_/FiO_2_ ratio represents one of the criteria for the score [16]. Other studies have tried to optimize the prognostic effectiveness of existing scores in patients hospitalized with COVID-19. An example is National Early Warning Score 2 Plus (NEWS 2 Plus) [17]. Furthermore, our research group has recently developed the highly performing prognostic Cascio–Zinna COVID-19 score (CZ COVID-19) [18].

The first purpose of this study was to assess the predictive accuracy of the principal risk scores used during the COVID-19 pandemic and to compare them in terms of predicting in-hospital mortality. Secondly, our study aimed to evaluate the role of the PaO_2_/FiO_2_ and SpO_2_/FiO_2_ ratios as independent predictors of mortality in admitted patients with COVID-19 pneumonia and their predictive utility when combined with other scores developed to assess the severity of pneumonia or COVID-19.

## 2. Materials and Methods

### 2.1. Study Design and Setting

This retrospective study was conducted at multiple departments of two Italian University hospitals during the first and second waves of the COVID-19 pandemic: the infectious and tropical disease unit, internal medicine unit, and geriatric unit of the University Hospital “Policlinico Paolo Giaccone” of Palermo (Italy), and the infectious and tropical disease unit of the “Luigi Vanvitelli” University Hospital of Campania (Italy).

The medical records of 230 consecutive patients over 18 years admitted to the University Hospital of Palermo (first cohort) and of 1357 consecutive patients over 18 years admitted to the “Luigi Vanvitelli” University Hospital of Campania (Italy) (second cohort) during the first and second wave of the COVID-19 pandemic, between June 2020 and December 2021, were analyzed. The data collected refers to the period when the treatment protocols were developed, and no standard treatment was available. Therefore, none of the patients had previously received “early therapy” with antivirals or monoclonal.

The diagnosis of COVID-19 was based on the positive respiratory tract real-time polymerase chain reaction (RT-PCR) results for the SARS-CoV-2 nucleic acid.

This study was conducted in accordance with the Declaration of Helsinki [19].

All the experiments were performed following the relevant guidelines and regulations. Informed consent was obtained from all participants and/or their legal guardians.

This study was approved by the Ethics Committee of the University of Campania L. Vanvitelli, Naples (n°10,877/2020) and the Ethics Committee “Palermo I” of the A.O.U. Policlinico “Paolo Giaccone”, Palermo, Italy (verbal n. 11/2022, 12 December 2022).

### 2.2. Data Collection

The following variables were extracted for the first cohort of patients admitted to the University Hospital “Policlinico Paolo Giaccone” of Palermo (Italy):

- epidemiological and demographic characteristics,
- medical comorbidities,
- Charlson Comorbidity Index (CCI) [20] on admission,
- days from onset of symptoms to hospitalization,
- days from positive swab to hospitalization,
- days from onset of symptoms to positive swab,
- Pneumonia Severity Index (PSI) [21] on admission,
- CRB-65 score, abbreviated for confusion, respiratory rate, BP, and age > 65 years [22,23] on admission,
- National Early Warning Score 2 (NEWS2) [24] on admission,
- International Severe Acute Respiratory Infection Consortium Coronavirus Clinical Characterisation Consortium (ISARIC 4C) [3,25] on admission,
- hospitalization or outpatient management of Patients with SARS-CoV-2 (HOME-CoV) [26] on admission,
- ABC2-SPH risk score (ABC2-SPH) [14,15] on admission,
- CAPS-D score [27] on admission,
- SOARS score, abbreviated for SpO_2_, obesity, age, respiratory rate, and stroke history (SOARS) [28] on admission,
- COVID-19 Severity Index [29] on admission,
- ASCL score, abbreviated for age, sex, CRP at hospital admission, and LDH at hospital admission (ASCL) [30] on admission,
- COVID-19 Early Warning Score (COEWS) [31] on admission,
- National Early Warning Score 2 Plus (NEWS2 Plus) [17] on admission,
- Cascio–Zinna COVID-19-mortality Score (CZ COVID-19) [18] on admission,
- clinical presentation,
- vital signs at first clinical contact (blood pressure, heart rate, oxygen saturation, respiratory rate, body temperature),
- respiratory function,
- laboratory tests,
- imaging,
- oxygen therapy,
- days of hospitalization.

For the second cohort, patients admitted to the Luigi Vanvitelli University Hospital in Campania (Italy), fewer variables but sufficient for our study were extracted. Unfortunately, the SpO_2_/FiO_2_ ratio could not be evaluated in the second cohort due to a lack of data.

The above data were derived from paper medical records and stored in a Microsoft Access database.

### 2.3. Statistical Analyses

Data distribution was evaluated using the Shapiro–Wilk test. In the descriptive analysis, the mean (μ) and standard deviation (SD) were calculated for continuous values with normal distribution and median (μe) and interquartile range (IQR) for continuous values without normal distribution, and absolute and relative frequencies were calculated for categorical variables.

In the bivariate analysis, we calculated *p*-values using an unpaired Student’s *t*-test and Chi-Square for categorical variables. A *p*-value of less than 0.05 was considered statistically significant. All tests were two-tailed.

Multivariable analysis was performed using forward conditional stepwise logistic regression to examine the association between clinical and laboratory parameters and the risk of death. The dependent variable was a favorable clinical outcome or death, and the independent variables were selected according to clinical and statistical criteria in several stages.

The PaO_2_/FiO_2_ ratio threshold chosen to plot the receiver operating characteristic (ROC) curves was ≤300 mmHg; this threshold corresponds to the definition of both severe COVID-19 and acute respiratory distress syndrome (ARDS) [32]. The SpO_2_/FiO_2_ ratio thresholds were determined using the ROC curves. The agreement between the SpO_2_/FiO_2_ and the PaO_2_/FiO_2_ ratios was assessed using the Pearson correlation coefficient [33].

Failure to register one of the parameters studied in this clinical study was the criteria for exclusion at entry into this study.

For the statistical analysis, we used IBM SPSS, version 26, and MedCalc and Matlab, version R2023b; for graphical statistical presentation, we used the GraphPad Prism Package, version 9, and TIBCO Software Statistica (formerly StatSoft), version 10.

### 2.4. Combination of Risk Scores and an Independent Predictor of Mortality

We examined the discriminative power of the PaO_2_/FiO_2_ and SpO_2_/FiO_2_ ratios in predicting mortality in patients with COVID-19 pneumonia and their predictive utility when combined with CCI, with CRB-65 and NEWS2 scores, and with new main scorings developed during the COVID-19 pandemic such as the ISARIC 4C, HOME-CoV, ABC2-SPH, CAPS-D, SOARS, COVID-19 Severity Index, ASCL, COEWS and CZ COVID-19. The combinations of these rating systems with independent predictors of mortality (PaO_2_/FiO_2_ or SpO_2_/FiO_2_ ratios) were formed by logistic regression analysis of the estimates of predicted probabilities. The ROC analysis was used to calculate the area under the curve (AUC) of the prediction models. DeLong’s statistic test was used to compare the AUC of the prediction models [34]. In the end, the net reclassification index (NRI) was calculated to measure the prediction increment of these new biomarkers, PaO_2_/FiO_2_ or SpO_2_/FiO_2_, when added to various prognostic scores.

## 3. Results

We included 230 patients (40 died, 17.39% of the sample) with a μ ± SD age of 66.91 ± 15.50 in the first cohort and 1357 patients (104 died, 7.67% of the sample) with a μ ± SD age of 61.33 ± 16.07 in the second cohort (Supplementary Table S1).

In the first cohort, the most common comorbidities encountered were arterial hypertension with 136 (59.1%) cases, diabetes (81, 35.2%), and cardiovascular disease (47, 20.4%); 105 patients (45.7%) had a CCI > 4 points, of these, 29 patients died (72.5%, *p* < 0.001). In the first cohort, the most frequent symptoms of clinical onset were fever (53.5%) and cough (25.2%); 67 (29.1%) and 60 patients (26.1%) had a CRB-65 on admission ≥ 2 points and a PSI on admission ≥ IV risk class, respectively; of these, ≥ 70% died (72.5%, *p* < 0.001; 70.0%, *p* < 0.001; respectively). In the same way, 84 (36.5%) and 125 patients (85.0%) had an ABC2-SPH on admission ≥ 5 points and SOARS on admission ≥ 3 points, respectively; of these, 72.5% (*p* < 0.001) and 85.0% died (*p* < 0.001), respectively. A total of 142 (61.7%) and 168 patients (73.0%) had a COVID-19 Severity Index on admission and ASCL on admission ≥ 7 points, respectively; of these, 30 (75.0%, *p* = 0.058) and 35 patients (87.5%, *p* = 0.023) died, respectively. Almost all deceased patients had a NEWS2 on admission ≥ 3 points (36 dead patients, 90.0%) and NEWS2 Plus on admission ≥ 5 points (40 dead patients, 100%). A total of 203 patients (88.3%) were classified in a high-risk group for CZ COVID-19; of these, 40 patients died (*p* < 0.001). The median calculated for ISARIC 4C on admission, COEWS on admission, and CAPS-D on admission were 6 points (IQR 5–7), 2 points (1–4) and 11 points (IQR 8–13), respectively, with an increase in the calculated score to 7 points (IQR 6–8, *p* = 0.002) in dead patients for ISARIC 4C, 14 points (IQR 35.0, *p* = 0.002) in dead patients for COEWS and 14 points (IQR 10–16, *p* < 0.001) in dead patients for CAPS-D. (Supplementary Table S1).

The mean duration of the hospital recovery ± SD was 15.94 ± 11.55 and 15.86 ± 9.21 days in the first and second cohorts, respectively (Supplementary Table S1).

**Figure 1:**
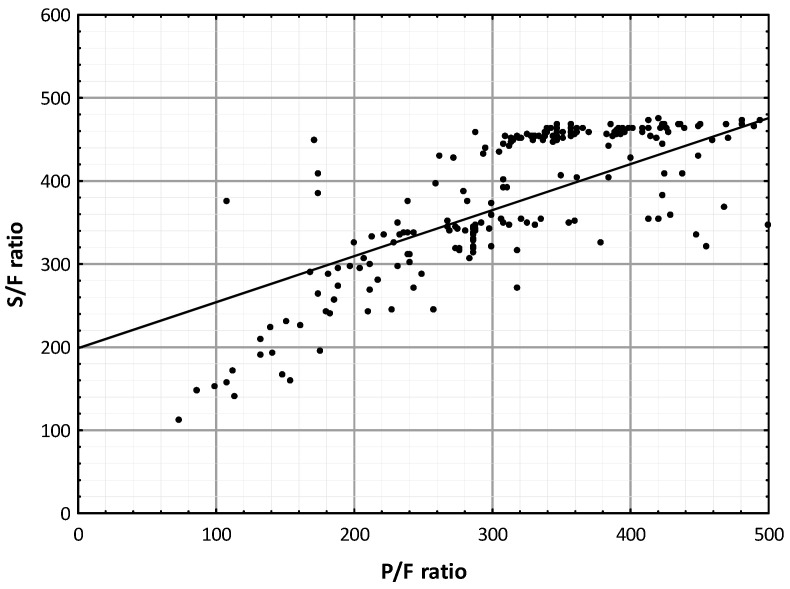
S/F ratio vs. *p*/F ratio scatter plot in the first cohort. The line represents the best fit linear relationship. Abbreviations: P/F, PaO_2_/FiO_2_ ratio; S/F, SaO_2_/FiO_2_ ratio. (*p* < 0.001 by Pearson correlation coefficient).

**Figure 2:**
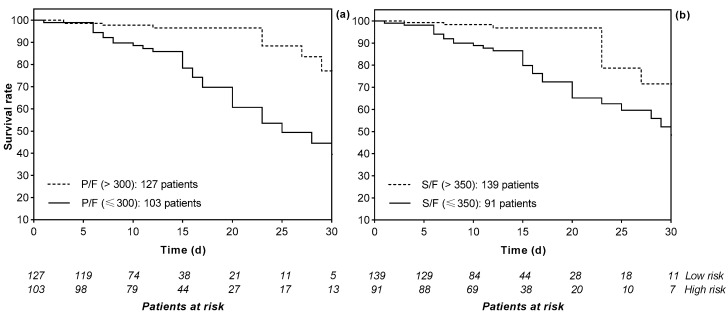
Survival curve according to the PaO_2_/FiO_2_ ratio (*p* < 0.001 by Log-rank test) (**a**) and the SpO_2_/FiO_2_ ratio (*p* < 0.001 by Log-rank test) (**b**) in the first cohort, respectively. Abbreviations: P/F, PaO_2_/FiO_2_ ratio; S/F, SaO_2_/FiO_2_ ratio.

The hemoglobin (Hb) values were lower in patients who died (11.05 ± 2.53 g/dL, *p* < 0.001, first cohort; 12.71 ± 1.14 g/dL, *p* = 0.05, second cohort), as were the platelet (PLT) count (208 ± 108 × 10^3^/µL, *p* = 0.003, first cohort; 289 ± 110 × 10^3^/µL, *p* = 0.174, second cohort), baseline oxygen saturation (SpO_2_) percentage (95 ± 3%, *p* = 0.041, first cohort; 85 ± 6%, *p* < 0.001, second cohort); conversely, the white blood cell (WBC) count values were higher in patients who died (18,417 ± 5595 cell/µL, *p* = 0.009, first cohort; 9722 ± 5008 cell/µL, *p* = 0.006, second cohort) (Supplementary Table S1). Likewise, the PaO_2_/FiO_2_ ratio (287 ± 70, *p* = 0.015, first cohort; 182 ± 94, *p* < 0.001, second cohort) and the SpO_2_/FiO_2_ ratio (353 ± 78, *p* = 0.046, first cohort) values were lower in patients who died (Supplementary Table S1). A scatter plot of the SpO_2_/FiO_2_ and PaO_2_/FiO_2_ ratios demonstrated a linear correlation in the first cohort (*p* < 0.001 by Pearson correlation coefficient) (Figure 1). A total of 103 (44.8%) and 595 patients (43.8%) had a PaO_2_/FiO_2_ ratio ≤ 300 on admission in the first and second cohorts; of these, >80% died (82.5%, *p* = 0.015; 92.9%; *p* < 0.001; respectively). In the first cohort, 91 patients (39.6%), of which 30 patients died (75.0%), had a SpO_2_/FiO_2_ ratio ≤ 350 (Supplementary Table S1). We classified the patients into two risk categories (a low-risk group and a high-risk group) according to their PaO_2_/FiO_2_ and SpO_2_/FiO_2_ ratios in the first cohort and only according to their PaO_2_/FiO_2_ ratio in the second cohort (*p* < 0.001 by Log-rank test) (Figure 2). Figure 2 shows the above groups’ Kaplan-Meier curve [35].

After performing multivariate analysis, only age (OR 1.130, 95% CI 1.127–1.186 and *p* < 0.001 in the first cohort; OR 1.117, 95% CI 1.093–1.142 and *p* < 0.001 in the second cohort) and the PaO_2_/FiO_2_ ratio (OR 0.994, 95% CI 0.989–0.999 and *p* = 0.022 in first cohort; OR 0.990, 95% CI 0.897–0.993 and *p* < 0.001 in second cohort) were found to be independent parameters in predicting mortality in both cohorts (Table 1).

### Comparison Scores and Combined Scores with the PaO_2_/FiO_2_ or SpO_2_/FiO_2_ Ratios

**Figure 3:**
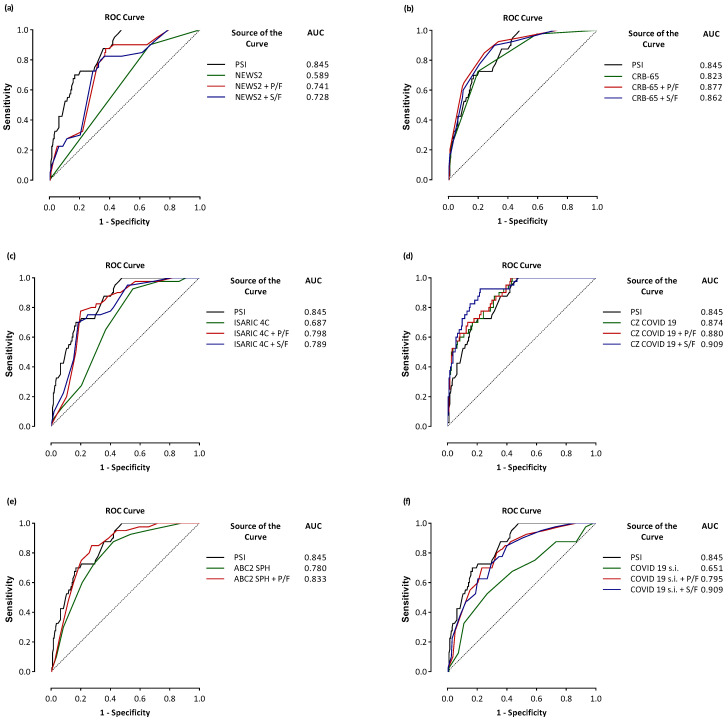

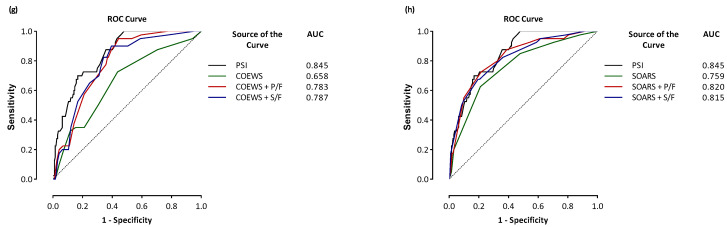
ROC curve of the risk scores in predicting mortality. Comparison between the PSI vs. other risk scores and the PSI vs. the combination of P/F or S/F with the other risk scores. Comparison between the PSI vs. NEWS2 (**a**), the PSI vs. CRB 65 (**b**), the PSI vs. ISARIC 4C (**c**), the PSI vs. CZ COVID-19 (**d**), the PSI vs. ABC2 SPH (**e**), the PSI vs. COVID-19 Severity Index (**f**), the PSI vs. COEWS (**g**), the PSI vs. SOARS (**h**). Abbreviations: ABC2 SPH, ABC2-SPH risk score; COEWS, COVID-19 Early Warning Score; COVID-19 s.i., COVID-19 Severity Index; CRB 65, CRB-65 score abbreviated for confusion, respiratory rate, BP, and age > 65 years; CZ COVID-19, Cascio–Zinna COVID-19 mortality Score; ISARIC 4C, International Severe Acute Respiratory Infection Consortium Coronavirus Clinical Characterisation Consortium; NEWS2, National Early Warning Score 2; PSI, Pneumonia Severity Index; SOARS, SOARS score abbreviated for SpO_2_, obesity, age, respiratory rate, and stroke history.

The PSI, CRB-65, and NEWS2 have fair discriminative ability. The PSI scored better than CRB-65 and NEWS2 in predicting mortality (AUC 0.766 vs. 0.763, *p* = 0.891; AUC 0.766 vs. 0.616, *p* = 0.003, respectively). Adding the PaO_2_/FiO_2_ ratio to CRB-65 improved the performance compared to CRB-65 alone (AUC 0.877 vs. 0.763, *p* < 0.001), and adding the SpO_2_/FiO_2_ ratio to CRB-65 improved the performance compared to CRB-65 alone (AUC 0.862 vs. 0.763, *p* < 0.001). Both the PaO_2_/FiO_2_ ratio combined with CRB-65 and the SpO_2_/FiO_2_ ratio combined with CRB-65 have a good discriminative ability and seemed to be more favorable than the PSI (AUC 0.877 vs. 0.766; AUC 0.862 vs. 0.766), and significant differences were found (*p* < 0.001 and *p* < 0.001, respectively) (Table 2 and Figure 3).

Similarly, adding the PaO_2_/FiO_2_ and SpO_2_/FiO_2_ ratios to NEWS2 improved the performance compared to NEWS2 alone (AUC 0.741 vs. 0.616, *p* < 0.001; AUC 0.728 vs. 0.616, *p* < 0.001; respectively). However, the PaO_2_/FiO_2_ or SpO_2_/FiO_2_ ratios combined with NEWS2 did not have a good discriminative ability and seemed to be more favorable than PSI (AUC 0.741 vs. 0.766; AUC 0.728 vs. 0.766; respectively), and no significant differences were found (*p* = 0.631 and *p* = 0.483, respectively) (Table 2 and Figure 3).

The results of the global comparative analysis of the ROC curves and Net Reclassification Improvement of risk scorings are shown in Table 3. Every score was compared to the PaO_2_/FiO_2_ or the SpO_2_/FiO_2_ ratios combined with corresponding scores. This initial examination revealed that combining the PaO_2_/FiO_2_ or SpO_2_/FiO_2_ ratios with the same score enhances predictive validity. The COWES score (AUC 0.658, 95% IC 0.593–0.719) is an example, compared to the PaO_2_/FiO_2_ ratio combined with COWES (AUC 0.783, 95% IC 0.724–0.834, *p* = 0.005; NRI 0.311, *p* = 0.016) and the SpO_2_/FiO_2_ ratio combined with COWES (AUC 0.787, 95% CI 0.729–0.983, *p* = 0.009; NRI 307, *p* = 0.012). The PSI was compared to the other scores with or without the PaO_2_/FiO_2_ or SpO_2_/FiO_2_ ratios in Table 3. The analysis shows that the PSI (AUC 0.845, 95% CI 792–0.890, 0.792–0.890) is similar to the CRB-65 (AUC 0.823, 95% CI 0.767–0.870, *p* = 0.294), SOARS (AUC 0.759, 95% CI 0.699–0.813, *p* = 0.045) and CZ COVID–19 (AUC 0.874, 95% CI 0.824–0.914, *p* = 0.427). The PaO_2_/FiO_2_ or SpO_2_/FiO_2_ ratios, if added to other scores with lower predictive power than the PSI, determined an increase in their diagnostic accuracy equal to the PSI, according to this study. The COVID-19 Severity Index (AUC 0.651, 95% CI 0.586–0.713, *p*-value compared to PSI 0.001) is an example. Indeed, the COVID-19 Severity Index combined with the PaO_2_/FiO_2_ ratio has an AUC of 0.795 (95% CI 0.737–0.845, *p*-value compared to PSI = 0.229), and it has an NRI 0.212 (*p* = 0.013). The same COVID-19 Severity Index combined with the SpO_2_/FiO_2_ ratio has an AUC of 0.909 (95% CI 0.864–0.943, *p*-value compared to PSI = 0.168), and it has an NRI 0.193 (*p* = 0.039). Similar findings were highlighted for other risk scores and ISARIC 4C (AUC 0.687, 95% CI 0.623–0.746, *p* < 0.001), ISARIC 4C combined with the PaO_2_/FiO_2_ ratio (AUC 798, 95% CI 0.740–0.848, *p* = 0.220; NRI 203, *p* = 0.006), and ISARIC 4C combined with the SpO_2_/FiO_2_ (AUC 0.789, 95% CI 0.730–0.839, *p* = 0.156; NRI 143, *p* = 0.043). Although not statistically significant (*p* = 0.068), the SpO_2_/FiO_2_ ratio with CZ COVID-19 has an AUC of 0.909 (95% CI 0.894–0.943), a sensitivity of 92.5%, and a specificity of 77.9% highest respect the PSI (AUC 0.845, sensitivity of 70.0%, and specificity 83.2%). We obtained a similar result (AUC 0.909 vs. 0.875, sensitivity 92.5% vs. 87.5%, and specificity 77%, respectively) when comparing the SpO_2_/FiO_2_ ratio with CZ COVID-19 and the SpO_2_/FiO_2_ ratio with the PSI. The significance level was 0.249 (Table 3 and Figure 3).

## 4. Discussion

Decision-making tactics in the COVID-19 patients’ approach to hospital admission were the focus of our research. Our study shows that at the beginning of the pandemic, they had prognostic scores that were valid in identifying patients who required more care, for example, the PSI. Our study also shows that other existing prognostic scores, such as the CRB-65 or those developed during the pandemic wave, such as ISARIC 4C, are excellent prognostic scores when combined with independent predictors of mortality, as the PaO_2_/FiO_2_ or SpO_2_/FiO_2_ ratios.

The COVID-19 challenge has spread a strong message to the world: the need to build a resilient and sustainable health system [36]. The timely detection of COVID-19 patients with a higher risk of mortality is essential to reduce mortality and better manage hospital resources [37]. COVID-19 ARDS is a severe, predictable complication of COVID-19 that requires early recognition and comprehensive management [38]. Our analysis shows a strong association between mortality from COVID-19 with advanced age and low PaO_2_/FiO_2_ and SpO_2_/FiO_2_ ratios. Observations from our retrospective study showed that SARS-CoV-2 patients with PaO_2_/FiO_2_ ratio levels lower than 300 and with SpO_2_/FiO_2_ ratio levels lower than 350 are at higher risk of in-hospital mortality than those with higher levels. The PaO_2_/FiO_2_ ratio is used to classify the severity of ARDS [39]. A PaO_2_/FiO_2_ ratio < 300 showed at least a two- to three-fold correlation to adverse outcomes; this may provide simple but straightforward targets for clinicians facing COVID-19 respiratory failure in a non-ICU setting [40].

The noninvasive parameter SpO_2_/FiO_2_ ratio was found to be correlated with the PaO_2_/FiO_2_ ratio in patients with COVID-19 pneumonia [9,41]. The SpO_2_/FiO_2_ ratio, calculated on the first days of hospitalization, is independently associated with mortality, has a predictive capacity for 28-day mortality, and is a good alternative to the PaO_2_/FiO_2_ ratio [42]. Furthermore, the SpO_2_/FiO_2_ ratio is a noninvasive prognostic marker that facilitates early adjustment for treatment, thus improving overall survival [43]. Moreover, it is undeniable that pulse oximeters are becoming increasingly widespread and can provide costless monitoring. Hence, replacing the PaO_2_/FiO_2_ ratio with the SpO_2_/FiO_2_ ratio may allow resource-limited facilities to diagnose acute respiratory failure objectively [13]. The SpO_2_/FiO_2_ ratio has already been included in some scores, an example of which is the ABC2-SPH developed by a Spanish research group [14,15].

Our analysis confirms that the PSI score is a good predictor for mortality in patients with COVID-19 pneumonia, but it is very complex and includes many variables. In the evaluation of COVID-19 pneumonia, we need scores that are quick and easy to use [44]. Our analysis shows that the CRB-65 score was a good alternative predictor for mortality in patients with COVID-19 pneumonia, and a combination of the PaO_2_/FiO_2_ or SpO_2_/FiO_2_ ratios with CRB-65 was a good equal alternative to the PSI score. In a meta-analysis, Chalmers JD et al. [45] confirmed that CURB-65 and CRB-65 have a predictive performance similar to the PSI [46]. In another meta-analysis, Zaki HA et al. [47] obtained CURB-65 respect PSI was slightly better in early mortality prediction and predicting admission to intensive care support [46]. Finally, in several clinical studies comparing CURB-65 to CRB-65, it emerges that CRB-65 was more sensitive as a predictor of death as well as guidance for hospitalization, and it is a more practical score since it does not use laboratory variables [23,47,48]. The NEWS2 score may not accurately reflect the severity of hypoxemia and lung injury [49]. According to this study, the PaO_2_/FiO_2_ or SpO_2_/FiO_2_ ratios, when added to other prognostic scores with lower predictive power than the PSI, determined an increase in their diagnostic acuity equal to the PSI.

Since the first SARS-CoV-2 infection was identified in December 2019, the COVID-19 pandemic has significantly increased morbidity and mortality around the world [50]. Early detection of patients likely to develop critical illness is essential and may aid in delivering proper care and optimizing the use of limited resources [51]. Several studies have derived prognostic predictors for COVID-19 [3]. It is important to use clear and objective criteria for risk stratification and early diagnosis of patients at a high risk of clinical worsening [52]. Thanks to this, new risk scores for predicting in-hospital mortality have been developed by categorizing patients at low, intermediate, high, or very high risk of death [3,16]. Prognostic scores attempt to transform complex clinical pictures into tangible numerical values [3]. Unfortunately, only a few models have found their way into routine care at the ED [53].

The ISARIC 4C mortality score indicates mortality risk at admission based on demographic and physiological parameters derived from a national-level population cohort study in the United Kingdom (UK) [54]. The CURB-65 and ISARIC 4C scores showed comparable performance [55]. The CZ COVID-19 score is a valid alternative to the PSI score for quickly stratifying COVID-19 patients on admission and facilitating decision-making. Furthermore, the SpO_2_/FiO_2_ ratio combined with the CZ COVID-19 score has a greater predictive power than the PSI.

This study looked at the role of the SpO_2_/FiO_2_ ratio as an independent factor linked to death, and it was seen that this biomarker, when added to the diagnostic scores for pneumonia and COVID-19, increases their accuracy. We used both the standard AUC metric and the NRI metric to accomplish this. The NRI is an increasingly popular measure for evaluating improvements in risk predictions [56]. Despite its limitations, the NRI seeks to quantify whether a new marker provides clinically relevant improvements in prediction models [57,58].

## 5. Conclusions

Adding the SpO_2_/FiO_2_ ratio to the traditional, validated, and already internationally known pre-pandemic prognostic scores is a valid alternative to the need for developing new prognostic scores. The SpO_2_/FiO_2_ ratio combined with the multiple scores developed for the COVID-19 disease significantly increases their predictive power. The SpO_2_/FiO_2_ ratio combined with the CZ COVID-19 score obtained a greater sensitivity and specificity than the PSI.

## Figures and Tables

**Table 1:** Multivariable model for predicting mortality in the two patient cohorts.

Variables	First Cohort (n: 230)		Second Cohort (n: 1357)	
	mOR (95% CI)	*p*-Value	mOR (95% CI)	*p*-Value
Age (years)	1.13 (1.127–1.186)	<0.001	1.117 (1.093–1.142)	<0.001
Hb (g/dL)	0.586 (0.462–0.743)	<0.001	1.173 (0.951–1.447)	0.128
WBC (cell/μL)	3.541 (1.305–9.607)	0.013	1.117 (0.682–1.830)	0.659
PLT (×10^3^/µL)	0.111 (0.036–0.345)	<0.001	1.581 (0.991–2.521)	0.054
P/F	0.994 (0.989–0.999)	0.022	0.990 (0.897–0.993)	<0.001

Abbreviations: P/F, PaO_2_/FiO_2_ ratio; 95% CI, 95% confidence interval; Hb, hemoglobin; mOR, multivariable odds ratio; n, number of patients; PLT, platelets; WBC, white blood cells.

**Table 2:** Diagnostic test performance of the risk scores in predicting the mortality of the 230 patients in the first cohort.

Value %	NEWS2 ≥ 3	NEWS2 + P/F n *	NEWS2 + S/F *	CRB-65 ≥ 2	CRB-65 + P/F *	CRB-65 + S/F *	PSI ≥ IV
Sensitivity	33.2	87.5	80.0	72.5	85.0	90.0	70.0
Specificity	90.0	62.5	65.8	80.0	75.8	68.9	83.2
NLR	2.0	0.6	0.5	0.3	0.3	0.5	0.2
PLR	9.0	7.0	4.0	2.6	5.6	9.0	2.3
NPV	36.4	33.0	33.0	43.3	42.5	37.9	46.7
PPV	22.1	96.0	94.0	93.3	96.0	97.0	92.9
95% CI	0.550–0.679	0.679–0.796	0.666–0.784	0.702–0.816	0.827–0.916	0.810–0.903	0.706–0.819
*p*-value	0.001	<0.001	<0.001	<0.001	<0.001	<0.001	<0.001
AUC	0.616	0.741	0.728	0.763	0.877	0.862	0.766
*p*-value **	Reference	<0.001	<0.001	Reference	<0.001	0.001	–
*p*-value ***	0.003	0.631	0.483	0.891	<0.001	0.005	Reference

Abbreviations: 95% CI, 95% confidence interval; AUC, area under the curve; CRB-65, CRB-65 score abbreviated for confusion, respiratory rate, BP, and age > 65 years; NEWS2, National Early Warning Score 2; NLR, negative likelihood ratio; NPV, negative predictive value; P/F, PaO_2_/FiO_2_ ratio; PLR, positive likelihood ratio; PPV, positive predictive value; PSI, Pneumonia Severity Index; S/F, SaO_2_/FiO_2_ ratio. * Considering the most appropriate cut-off value. ** Comparison of DeLong’s test *p*-value between reference scores and the PaO_2_/FiO_2_ or SpO_2_/FiO_2_ combined with same scores. *** Comparison of DeLong’s test *p*-value between PSI and other scores.

**Table 3:** Comparative analysis of the ROC curves and Net Reclassification Improvement of risk scores by adding the PaO_2_/FiO_2_ or SpO_2_/FiO_2_ ratios applied to the 230 patients in the first cohort.

Scores	Year of Birth	Nation of Birth	Sensitivity (%)	Specificity (%)	AUC	95% CI	*p*-Value	*p*-Value ^$^	*p*-Value ^$$^	*p*-Value	NRI (*p*-Value)
CCI	1987	USA	72.5	60.0	0.685	0.621–0.745	<0.001	Reference	0.001	–	Reference
CCI + P/F *			75.0	78.4	0.807	0.750–0.856	<0.001	0.001	0.398	0.012 ^$$$^	0.210 (1.613)
CCI + S/F *			72.5	79.5	0.786	0.727–0.837	<0.001	0.007	0.216	0.009 ^$$$$^	0.194 (1.181)
PSI	1998	USA	70.0	83.2	0.845	0.792–0.890	<0.001	Reference	Reference	–	Reference
PSI + P/F *			90.0	78.4	0.887	0.838–0.924	<0.001	0.052	–	Reference ^$$$^	0.153 (0.035)
PSI + S/F *			87.5	75.8	0.875	0.825–0.915	<0.001	0.162	–	Reference ^$$$$^	0.101 (0.142)
CRB-65	2002	UK	72.5	80.0	0.823	0.767–0.870	<0.001	Reference	0.294	–	Reference
CRB-65 + P/F *			85.0	75.8	0.877	0.827–0.916	<0.001	0.023	0.253	0.548 ^$$$^	0.083 (0.152)
CRB-65 + S/F *			90.0	68.9	0.862	0.810–0.903	<0.001	0.019	0.572	0.057 ^$$$$^	0.039 (0.548)
NEWS2	2017	UK	90.0	33.2	0.589	0.523–0.653	0.061	Reference	<0.001	–	Reference
NEWS2 + P/F *			87.5	62.6	0.741	0.679–0.796	<0.001	<0.001	0.020	<0.001 ^$$$^	0.195 (<0.001)
NEWS2 + S/F *			80.0	65.8	0.728	0.666–0.784	<0.001	<0.001	0.012	0.012 ^$$$$^	0.226 (<0.001)
ISARIC 4C	2020	UK	92.5	44.7	0.687	0.623–0.746	<0.001	Reference	<0.001	–	Reference
ISARIC 4C + P/F *			77.5	80.0	0.798	0.740–0.848	<0.001	0.003	0.220	0.001 ^$$$^	0.203 (0.006)
ISARIC 4C + S/F *			70.0	81.6	0.789	0.730–0.839	<0.001	0.005	0.156	0.254 ^$$$$^	0.143 (0.043)
HOME-CoV	2020	FR	90.0	40.5	0.666	0.601–0.727	<0.001	Reference	<0.001	–	Reference
HOME-CoV + P/F *			75.0	75.3	0.777	0.717–0.829	<0.001	0.002	0.097	<0.001 ^$$$^	0.223 (0.002)
HOME-CoV + S/F *			85.0	60.0	0.787	0.729–0.838	<0.001	0.006	0.078	0.028 ^$$$$^	0.405 (2.887)
ABC2-SPH	2021	ES	87.5	57.9	0.780	0.721–0.832	<0.001	Reference	0.116	0.018 ^$$$$^	Reference
ABC2-SPH + P/F *			85.0	72.6	0.833	0.778–0.879	<0.001	0.040	0.745	0.041 ^$$$^	0.109 (0.043)
CAPS-D	2021	GER	57.5	83.2	0.748	0.687–0.803	<0.001	Reference	0.048	–	Reference
CAPS-D + P/F *			85.0	69.5	0.813	0.756–0.861	<0.001	0.058	0.440	0.016 ^$$$^	0.364 (0.007)
CAPS-D + S/F *			82.5	72.6	0.810	0.753–0.858	<0.001	0.085	0.396	0.039 ^$$$$^	0.428 (0.001)
SOARS	2021	UK	62.5	78.9	0.759	0.699–0.813	<0.001	Reference	0.045	–	Reference
SOARS + P/F *			72.5	78.4	0.820	0.765–0.868	<0.001	0.003	0.527	0.029 ^$$$^	0.138 (0.040)
SOARS + S/F *			67.5	80.5	0.815	0.759–0.863	<0.001	0.005	0.454	0.036 ^$$$$^	0.214 (0.007)
COVID-19 sever index	2021	ARG	52.5	73.2	0.651	0.586–0.713	0.002	Reference	<0.001	–	Reference
COVID-19 Severity Index + P/F *			80.0	66.8	0.795	0.737–0.845	<0.001	0.002	0.229	0.001 ^$$$^	0.212 (0.013)
COVID-19 Severity Index + S/F *			92.5	77.9	0.909	0.864–0.943	<0.001	0.005	0.168	0.002 ^$$$$^	0.193 (0.039)
ASCL	2022	ITA	82.5	43.7	0.669	0.604–0.729	<0.001	Reference	0.002	–	Reference
ASCL + P/F *			85.0	63.2	0.783	0.725–0.835	<0.001	0.005	0.158	0.001 ^$$$^	0.093 (0.003)
ASCL + S/F *			75.0	70.0	0.782	0.723–0.834	<0.001	0.010	0.151	0.004 ^$$$$^	0.275 (<0.001)
COEWS	2023	m	72.5	56.3	0.658	0.593–0.719	0.001	Reference	0.001	–	Reference
COEWS + P/F *			95.0	55.8	0.783	0.724–0.834	<0.001	0.005	0.148	0.001 ^$$$^	0.311 (0.016)
COEWS + S/F *			85.0	60.0	0.787	0.729–0.838	<0.001	0.009	0.129	0.003 ^$$$$^	0.307 (0.012)
NEWS2 Plus	2024	TH	85.0	42.1	0.640	0.575–0.702	0.001	Reference	<0.001	–	Reference
NEWS2 Plus + P/F *			85.0	66.8	0.766	0.705–0.819	<0.001	0.001	0.063	0.001 ^$$$^	0.395 (3.289)
NEWS2 Plus + S/F *			80.0	67.4	0.752	0.691–0.806	<0.001	0.002	0.035	0.001 ^$$$$^	0.300 (<0.001)
CZ COVID-19	2024	ITA	100	56.3	0.874	0.824–0.914	<0.001	Reference	0.427	–	Reference
CZ COVID-19 + P/F *			100	57.4	0.880	0.831–0.919	<0.001	0.011	0.333	0.842 ^$$$^	0.011 (0.157)
CZ COVID-19 + S/F *			92.5	77.9	0.909	0.864–0.943	<0.001	0.036	0.068	0.249 ^$$$$^	0.160 (<0.001)

Abbreviations: 95%CI, 95% confidence interval; ABC2-SPH, ABC2-SPH risk score; ASCL, ASCL score abbreviated for age, sex, CRP at hospital admission, and LDH at hospital admission; AUC, area under the curve; CAPS-D, CAPS-D score; CCI, Charlson Comorbidity Index; COEWS, COVID-19 Early Warning Score; COVID-19 Severity Index, COVID-19 Severity Index; CRB-65, CRB-65 score abbreviated for confusion, respiratory rate, BP, and age > 65 years; CZ COVID-19, Cascio–Zinna COVID-19 mortality Score; USA, United States of America; UK, United Kingdom; FR, France; ES, Spain; GER, Germany; ARG, Argentina; m, multicontinental retrospective study; TH, Thailand; ITA, Italy; HOME-CoV score, hospitalization or outpatient management of patients with SARS-CoV-2 infection; ISARIC 4C, International Severe Acute Respiratory Infection Consortium Coronavirus Clinical Characterisation Consortium; NEWS2 Plus, National Early Warning Score 2 Plus; NEWS2, National Early Warning Score 2; NRI, Net Reclassification Improvement; P/F, PaO_2_/FiO_2_ ratio; PSI, Pneumonia Severity Index; S/F, SaO_2_/FiO_2_ ratio; SOARS, SOARS score abbreviated for SpO_2_, obesity, age, respiratory rate, AND stroke history. ^$^ Comparison of DeLong’s test *p*-value between the reference scores and PaO_2_/FiO_2_ or SpO_2_/FiO_2_ combined with other scores. ^$$^ Comparison of DeLong’s test *p*-value between “PSI score” and other scores. ^$$$^ Comparison of DeLong’s test *p*-value between “PSI score + ratio P/F” and “other scores + ratio P/F”. ^$$$$^ Comparison of DeLong’s test *p*-value between “PSI score + ratio S/F” and “other scores + ratio S/F”. * The prognostic scores were combined with PaO_2_/FiO_2_ or SpO_2_/FiO_2_ ratios.

## Data Availability

Data presented in this study are available on request from the corresponding author.

## Supplementary Materials

The following supporting information can be downloaded at: https://www.mdpi.com/article/10.3390/jcm13195884/s1, Table S1: Univariate and bivariate statistical analysis of the first and second cohort of patients.

## Acronyms and Abbreviations

ABC2-SPH: ABC2-SPH risk score; ASCL: ASCL score abbreviated for age, sex, CRP at hospital admission, and LDH at hospital admission; ARDS: acute respiratory distress syndrome; AUC: area under the curve; CAPS-D: CAPS-D score; CCI: Charlson Comorbidity Index; COEWS: COVID-19 Early Warning Score; COVID-19 Severity Index: COVID-19 Severity Index; CRB-65: CRB-65 score abbreviated for confusion, respiratory rate, BP, and age > 65 years; CZ COVID-19: Cascio–Zinna COVID-19 mortality score; FiO_2_: fraction of inspired O_2_; Hb: hemoglobin; HOME-CoV score: hospitalization or outpatient management of patients with SARS-CoV-2 infection; HR: heart rate; IL-6: interleukin-6; IMV: Invasive Mechanical Ventilation; IQR: interquartile range; ISARIC 4C: International Severe Acute Respiratory Infection Consortium Coronavirus Clinical Characterisation Consortium; NEWS2 Plus: National Early Warning Score 2 Plus; NEWS2: National Early Warning Score 2; NIV: non-invasive ventilation; NRI: Net Reclassification Index; LDH, lactate dehydrogenase; PLT, platelets; PSI, Pneumonia Severity Index; SD, standard deviation; SOARS: SOARS score abbreviated for SpO_2_, obesity, age, respiratory rate, and stroke history; SpO_2_: oxygen saturation; WBC: white blood cells; μ, mean; μe, median.
