# Supplementary material for: The SpO2/FiO2 Ratio Combined with Prognostic Scores for Pneumonia and COVID-19 Increases Their Accuracy in Predicting Mortality of COVID-19 Patients
Source: J Clin Med. 2024 Oct 2;13(19):5884. doi: 10.3390/jcm13195884 (PMC11478206; doi:10.3390/jcm13195884)
Supplement: Supplementary file 1 [file jcm-13-05884-s001.zip › jcm-3141392-supplementary.pdf]

Supplementary Table S1. Demographics, comorbidities, calculated scores on admission to hospital, symptoms and signs clinical onset, laboratory test, respiratory function, hospital oxygen therapy by clinical status on admission, time passed, and clinical outcome. Bivariate analysis according to status at discharge. For each variable, the total number of valid data is given in the first column. For each outcome, the absolute frequency, the percentage of the outcome, and the *p* Values for the comparison between alive and dead at discharge are given.

**Variables**	**Vital Status at Discharge**			***p value***
	**All patients**	**Alive**	**Dead**	
First cohort	n: 230	n: 190	n: 40	
Second cohort*	n: 1357*	n: 1253*	n:104*	
**Demographics**				
**Age**				
Age (n: 230) (μ±SD)	66.91 ± 15.61	64.29 ± 15.10	79.35 ± 11.54	<0.001
Age > 65 years (n: 230) (%)	138 (60.0)	101 (53.2)	37 (92.5)	<0.001
Age (n: 1357) (μ±SD)*	61.33 ± 16.07	59.92 ± 15.50	78.37 ± 12.74	0.001
Age > 65 years (n: 1357) (%)*	595 (43.8)	508 (40.5)	87 (83.7)	<0.001
**Sex**				
Sex Male (n: 230) (%)	133 (57.8)	114 (60.0)	19 (47.5)	0..146
Sex Female (n: 230) (%)	97 (42.2)	76 (40.0)	21 (52.5)	
Sex Male (n: 1357) (%)*	826 (60.8)	770 (61.5)	56 (53.8)	0.127
Sex Female (n: 1357) (%)*	531 (39.1)	483 (38.5)	48 (46.2)	
**Comorbidities**				
Mute past medical history (n: 230) (%)	20 (8.7)	19 (10.0)	1 (2.5)	0.126
Diabetes (n: 230) (%)	81 (35.2)	65 (34.2)	16 (40.0)	0.486
Obesity (n: 230) (%)	32 (13.8)	30 (15.8)	2 (5.0)	0.453
Hypertension (n: 230) (%)	136 (59.1)	110 (57.9)	26 (65.0)	0.406
COPD (n: 230) (%)	22 (1.3)	16 (8.4)	6 (15.0)	0.199
Asthma (n: 230) (%)	3 (1.3)	3 (1.6)	0 (0.0)	0.424
TB (n: 230) (%)	1 (0.4)	0 (0.0)	1 (2.5)	0.029
Cardiovascular disease (n: 230) (%)	47 (20.4)	34 (17.9)	13 (32.5)	0.037
Kidney disease (n: 230) (%)	23 (10.0)	16 (8.4)	7 (17.5)	0.088
CCI on admission (n: 230) (μe and IQR)	4 (3–6)	4 (2–6)	5 (4–7)	<0.001
CCI ≤ 4 (n: 230) (%), pt. low and intermediate risk	125 (54.3)	114 (60.0)	11 (27.5)	Reference
CCI >4 (n: 230) (%), pt. high risk	105 (45.7)	76 (40.0)	29 (72.5)	<0.001
**Symptoms clinical onset**				
Fever (n: 230) (%)	123 (53.5)	105 (55.3)	18 (45.0)	0.237
Cough (n: 230) (%)	58 (25.2)	56 (29.5)	2 (5.0)	0.001
Sputum (n: 230) (%)	4 (1.7)	3 (1.6)	1 (2.5)	0.685
Asthenia (n: 230) (%)	47 (20.4)	40 (21.1)	7 (17.5)	0.613
Dyspnea (n: 230) (%)	99 (43.0)	84 (44.2)	15 (37.5)	0.436
Anorexia (n: 230) (%)	1 (0.4)	1 (0.5)	0 (0.0)	0.646
Myalgia (n: 230) (%)	11 (4.8)	9 (4.7)	2 (5.0)	0.943
Arthalgia (n: 230) (%)	15 (6.5)	14 (7.4)	1 (2.5)	0.257
Loss of smell (n: 230) (%)	3 (1.3)	3 (1.6)	0 (0.0)	0.424
Loss of taste (n: 230) (%)	4 (1.7)	4 (2.1)	0 (0.0)	0.355
Diarrhea (n: 230) (%)	20 (8.7)	19 (10.0)	1 (2.5)	0.126
Vomit (n: 230) (%)	13 (5.7)	11 (5.8)	2 (5.0)	0.844
Headache (n: 230) (%)	9 (3.9)	9 (4.7)	0 (0.0)	0.160
Abdominal pain (n: 230) (%)	13 (5.7)	12 (6.3)	1 (2.5)	0.342
Gastrointestinal bleeding (n: 230) (%)	12 (5.2)	12 (6.3)	0 (0.0)	0.103
**Scores on admission (μe and IQR)**				
PSI (n: 230)	75 (57–92)	69 (54–86)	108 (82–131)	< 0.001
PSI class I-III (n: 230) (%), pt. low and intermediate risk	170 (73.9)	158 (83.2)	12 (30.0)	Reference
PSI class IV-V (n: 230) (%), pt. high risk	60 (26.1)	32 (16.8)	28 (70.0)	<0.001
CRB-65 (n: 230)	1 (1–2)	1 (1–2)	2 (1–2)	< 0.001
CRB-65 < 2 (n: 230) (%), pt. low and intermediate risk	163 (70.9)	152 (80.0)	11 (27.5)	Reference
CRB-65 ≥ 2 (n: 230) (%), pt. high risk	67 (29.1)	38 (20.0)	29 (72.5)	<0.001
NEWS2 (n: 230)	3 (2–5)	4 (2–5)	3 (3–7)	0.007
NEWS2 < 3 (n: 230) (%), pt. low and intermediate risk	67 (29.1)	63 (33.2)	4 (10.0)	Reference
NEWS2 ≥ 3 (n: 230) (%), pt. high risk	163 (70.9)	127 (66.8)	36 (90.0)	0.003
ISARIC 4 C (n: 230)	6 (5–7)	6 (4–7)	7 (6–8)	0.002
ISARIC 4 C < 9 (n: 230) (%), pt. low and intermediate risk	211 (91.7)	176 (92.6)	35 (87.5)	Reference
ISARIC 4 C ≥ 9 (n: 230) (%), pt. high risk	19 (8.3)	14 (7.4)	5 (12.5)	0.284
HOME-CoV (n: 230)	1 (1–2)	1 (1–1)	1 (1–2)	0.075
HOME-CoV ≤ 3 (n: 230) (%), pt. low and intermediate risk	226 (98.3)	187 (98.4)	39 (97.5)	Reference
HOME-CoV > 3 (n: 230) (%), pt. high risk	4 (1.7)	3 (1.6)	1 (2.5)	0.685
ABC2-SPH (n: 230)	4 (1–6)	3 (1–5)	6 (4–7)	<0.001
ABC2-SPH < 5 (n: 230) (%), pt. low and intermediate risk	146 (63.5)	135 (71.1)	11 (27.5)	Reference
ABC2-SPH ≥ 5 (n: 230) (%), pt. high risk	84 (36.5)	55 (28.9)	29 (72.5)	<0.001
CAPS-D (n: 230)	11 (8–13)	10 (8–12)	14 (10–16)	<0.001
CAPS-D < 17 (n: 230) (%), pt. low and intermediate risk	213 (92.6)	180 (94.7)	33 (82.5)	Reference
CAPS-D ≥ 17 (n: 230) (%), pt. high risk	17 (7.4)	10 (5.3)	7 (17.5)	0.007

SOARS (n: 230)	3 (1–4)	2 (1–3)	4 (3–4)	<0.001
SOARS < 3 (n: 230) (%), pt. low and intermediate risk	105 (45.7)	99 (52.1)	6 (15.0)	Reference
SOARS ≥ 3 (n: 230) (%), pt. high risk	125 (54.3)	91 (47.9)	34 (85.0)	<0.001
COVID-19 severity index (n: 230)	7 (6–9)	7 (5–9)	9 (6–10)	0.010
COVID-19 severity index < 7 (n: 230) (%), pt. low and intermediate risk	88 (38.3)	78 (41.1)	10 (25.0)	Reference
COVID-19 severity index ≥ 7 (n: 230) (%), pt. high risk	142 (61.7)	112 (58.9)	30 (75.0)	0.058
ASCL (n: 230)	9 (6–11)	8 (6–11)	11 (8–12)	0.008
ASCL < 7 (n: 230) (%), pt. low and intermediate risk	62 (27.0)	57 (30.0)	133 (70.0)	Reference
ASCL ≥ 7 (n: 230) (%), pt. high risk	168 (73.0)	5 (12.5)	35 (87.5)	0.023
COEWS (n: 230)	2 (1–4)	2 (1–4)	4 (2–7)	0.002
COEWS < 6 (n: 230) (%), pt. low and intermediate risk	187 (81.3)	161 (84.7)	26 (65.0)	Reference
COEWS ≥ 6 (n: 230) (%), pt. high risk	43 (18.7)	29 (15.3)	14 (35.0)	0.004
NEWS2 Plus (n: 230)	7 (6–9)	7 (5–8)	7 (7–11)	0.044
NEWS2 Plus < 5 (n: 230) (%), pt. low and intermediate risk	33 (14.3)	33 (17.4)	0 (0.0)	Reference
NEWS2 Plus ≥ 5 (n: 230) (%), pt. high risk	197 (85.7)	157 (82.6)	40 (100)	0.004
CZ COVID-19 (n: 230)	−4 ((−6) −(−1))	−3 ((−5) − 0)	−8 ((−10) −(−6))	<0.001
CZ COVID-19 > 1 (n: 230) (%), pt. low and intermediate risk	27 (11.7)	27 (14.2)	0 (0.0)	Reference
CZ COVID-19 ≤ 1 (n: 230) (%), pt. high risk	203 (88.3)	163 (85.8)	40 (100)	0.011
**Laboratory test**				
Hb (g/dL) (n: 230) (μ±SD)	12.65 ± 2.19	12.98 ± 1.96	11.05 ± 2.53	<0.001
Hb (g/dL) (n: 1357) (μ±SD)*	13.72 ± 1.25	13.95 ± 1.14	12.71 ± 1.25	0.050
WBC (cell/μL) (n: 230) (μ±SD)	10,135 ± 22,302	8391 ± 3862	18,417 ± 5565	0.009
WBC (cell/μL) (n: 1357) (μ±SD)*	8701 ± 3971	8615 ± 3862	9722 ± 5008	0.006
Number of neutrophils (cell/μL) (n: 230) (μ±SD)	6663 ± 4448	6247 ± 3915	8640 ± 6085	0.002
Number of neutrophils (cell/μL) (n: 1357) (μ±SD)*	7070 ± 3668	6993 ± 3602	8496 ± 4516	0.001
Percentage of neutrophils (n: 230) (μ±SD)	75.15 ± 14.39	74.54 ± 13.99	78.08 ± 16.01	0.158
Percentage of neutrophils (n: 1357) (μ±SD)*	1048 ± 903	1056 ± 896	901 ± 1022	0.162
Number of lymphocytes (n: 230) (cell/μL)	1206 ± 723	1224 ± 720	1120 ± 735	0.407
Number of lymphocytes (n: 1357) (cell/μL)*	79.3 ± 9.49	79.0 ± 11.42	84.38 ± 11.38	<0.001
Percentage of lymphocytes (n: 230) (μ±SD)	16.71 ± 11.74	16.89 ± 10.65	15.89 ± 16.13	0.629
Percentage of lymphocytes (n: 1357) (μ±SD)*	13.8 ± 9.46	13.9 ± 96	10.18 ± 10.28	<0.001
PLT ($x10^3$/μL) (n: 230) (μ±SD)	249 ± 94	257 ± 89	208 ± 108	0.003
PLT ($x10^3$/μL) (n: 1357) (μ±SD)*	291 ± 110	305 ± 106	289 ± 110	0.174
Creatinine (mg/dL) (n: 230) (μ±SD)	1.20 ± 1.77	1.13 ± 1.81	1.57 ± 1.53	0.158
LDH (U/L) (n: 230) (μ±SD)	278 ± 99	278 ± 101	281 ± 97	0.894
PT/INR (n: 230) (μ±SD)	1.92 ± 9.31	1.96 ± 10.21	1.80 ± 1.65	0.924
aPTT (seconds) (n: 230) (μ±SD)	29.71 ± 14.93	29.59 ± 15.71	30.26 ± 10.68	0.796
Fibrinogen (mg/dL) (n: 230) (μ±SD)	541 ± 167	546 ± 161	517 ± 192	0.319
D-Dimer (ng/ml EFU) (n: 230) (μ±SD)	3019 ± 7615	2727 ± 7388	4406 ± 8579	0.209
CRP (mg/L) (n: 230) (μ±SD)	97.52 ± 583.70	53.69 ± 52.95	308.41 ± 1398.69	0.015
PCT (μg/L) (n: 230) (μ±SD)	1.66 ± 9.86	2.06 ± 11.71	0.71 ± 0.69	0.469
IL-6 (pg/mL) (n: 230) (μ±SD)	46.33 ± 64.61	39.78 ± 68.71	65.33 ± 46.63	0.031
Triglycerides (mg/dL) (n: 230) (μ±SD)	140 ± 55	148 ± 71	129 ± 12	0.088
Ferritin (ng/mL) (n: 230) (μ±SD)	806.90 ± 762.88	770.33 ± 9869.51	854.45 ± 604.32	0.603
Troponin (mg/L) (n: 230) (μ±SD)	290.23 ± 953.18	327.78 ± 1031.27	74.27 ± 44.39	0.633
BNPT (pg/mL) (n: 230) (μ±SD)	1467 ± 1948	1435 ± 2722	1,494 ± 961	0.897
**Respiratory function**				
Acts breath/minute (n: 230) (μ±SD)	19 ± 4	18 ± 5	20 ± 3	0.132
HR (n: 230) (μ±SD)	84 ± 14	84 ± 14	84 ± 10	0.929
Baseline SpO2 (n: 230) (μ±SD)	96 ± 3	96 ± 3	95 ± 3	0.041
Baseline SpO2 (n: 1357) (μ±SD)*	88 ± 7	89 ± 7	85 ± 6	<0.001
FiO_2 (n: 230) (μ±SD)	0.27 ± 0.09	0.27 ± 0.10	0.28 ± 0.06	0.557
PaO_2/FiO_2 (n: 230) (μ±SD)	326 ± 108	332 ± 113	287 ± 70	0.015
P/F >300 (n: 230) (%)	127 (55.2)	120 (63.2)	7 (17.5)	Reference
P/F ≤300 (n: 230) (%)	103 (44.8)	70 (36.8)	33 (82.5)	<0.001
PaO_2/FiO_2 (n: 1357) (μ±SD)*	248 ± 94	256±93	182±94	<0.001
P/F >300 (n: 1357) (%)*	762 (56.1)	347 (27.0)	5 (7.1)	Reference
P/F ≤300 (n: 1357) (%)*	595 (43.8)	940 (73.0)	65 (92.9)	<0.001
SpO_2/FiO_2 (n: 230) (μ±SD)	379 ± 87	383 ± 88	353 ± 78	0.046
S/F >350 (n: 230) (%)	139 (60.4)	129 (67.9)	10 (25.0)	Reference
S/F ≤350 (n: 230) (%)	91 (39.6)	61 (32.1)	30 (75.0)	<0.001
PaO_2st/FiO_2 (n: 230) (μ±SD)	308 ± 103	311 ± 109	292 ± 56	0.286
pH (n: 230) (μ±SD)	7.44 ± 0.05	7.44 ± 0.04	7.44 ± 0.07	0.916
Pa CO_2 mmHg (n: 230) (μ±SD)	36.78 ± 4.94	36.67 ± 4.99	37.21 ± 4.70	0.547
Pa O_2 mmHg (n: 230) (μ±SD)	82.06 ± 20.51	82.88 ± 21.19	78.11 ± 16.49	0.180
**Hospital oxygen therapy**				
Breathe in ambient air (n: 230) (%)	106 (46.1)	90 (47.7)	16 (40.0)	0.395
Nasal cannulas (n: 230) (%)	51 (22.2)	43 (22.6)	8 (20.0)	0.716
Facial mask (n: 230) (%)	11 (4.8)	7 (3.7)	4 (10.0)	0.089
Venturi mask (n: 230) (%)	58 (25.2)	46 (24.2)	12 (30.0)	0.443
NIV (n: 230) (%)	1 (0.4)	1 (0.5)	0 (0.0)	0.646
IMV (n: 230) (%)	3 (1.3)	3 (1.6)	0 (0.0)	0.547
**Time passed**				

Days of hospitalization (n: 230) (μ±SD)	15.94 ± 11.55	15.58 ± 11.48	17.63 ± 11.91	0.311
Days of hospitalization (n: 1357) (mean±SD)*	15.86 ± 9.21	16.16 ± 9.15	12.24 ± 9.23	<0.001
Days from symptom onset to Hospitalization (n: 230) (μ±SD)	7.35 ± 6.10	7.45 ± 6.01	6.88 ± 6.61	0.591
Days from positive swab to hospitalization (n: 230) (μ±SD)	2.92 ± 4.86	4.21 ± 5.73	4.75 ± 7.63	0.202
Days from symptom onset to positive swab (n: 230) (μ±SD)	4.30 ± 6.09	4.26 ± 2.77	5.75 ± 2.37	0.611
**Clinical outcome**				
Discharged home (n: 230) (%)	149 (64.8)	149 (78.4)	–	<0.001
Transferred to Covid Hotel (n: 230) (%)	30 (13.0)	30 (15.8)	–	0.007
Transferred to another department (n: 230) (%)	11 (4.8)	11 (5.8)	–	0.119

Abbreviation: ABC2-SPH, ABC2-SPH risk score; aPTT, activated partial thromboplastin time; ASCL, ASCL score abbreviated for age, sex, CRP at hospital admission, and LDH at hospital admission; BNPT, B-type natriuretic peptide; CAPS-D, CAPS-D score; CCI, Charlson Comorbidity Index; COEWS, COVID-19 Early Warning Score; COPD, chronic obstructive pulmonary disease; COVID-19 severity index, COVID-19 severity index; CRB-65, CRB-65 score; CRP, C-reactive protein; CZ COVID-19, Cascio-Zinna COVID-19 mortality Score; FiO_2: fraction of inspired O_2; Hb, hemoglobin; HOME-CoV score, Hospitalisation or Outpatient Management of patients with SARS-CoV-2 infection; HR, heart rate; IL-6, interleukin-6; IMV: Invasive Mechanical Ventilation; IQR, Interquartile range; ISARIC 4C, International Severe Acute Respiratory Infection Consortium-Coronavirus Clinical Characterisation Consortium; LDH, lactate dehydrogenase; n, number of patients; NEWS2 Plus, National Early Warning Score 2 Plus; NEWS2, National Early Warning Score 2; NEWS2, National Early Warning Score 2; NIV: Non–invasive ventilation; P/F, PaO_2/FiO_2 ratio; Pa, arterial partial pressure; PaO_2St, standardized arterial partial pressure oxygen; PCT, procalcitonin; pH, potential hydrogen; PLT, platelets; PSI, Pneumonia Severity Index; PT/INR, Prothrombin Time/International Normalized Ratio; pt., patients; S/F, SpO_2/FiO_2 ratio; SD, standard deviation; SOARS, SOARS score abbreviated for SpO_2, Obesity, Age, Respiratory rate, Stroke history; SpO_2, oxygen saturation; TB, tuberculosis; WBC, white blood cells; μ, mean; μe, median. * Second cohort.
